# Epidemiological and time series analysis of haemorrhagic fever with renal syndrome from 2004 to 2017 in Shandong Province, China

**DOI:** 10.1038/s41598-019-50878-7

**Published:** 2019-10-10

**Authors:** Chao Zhang, Xiao Fu, Yuanying Zhang, Cuifang Nie, Liu Li, Haijun Cao, Junmei Wang, Baojia Wang, Shuying Yi, Zhen Ye

**Affiliations:** 1 College of Basic Medicine, Shandong First Medical University & Shandong academy of medical sciences, Tai’an, 271016 China; 2 College of Information Science and Engineering, College of Information Science and Engineering Shandong Agricultural University, Tai’an, 271000 China; 3 Clinical Skills Training Center, Shandong First Medical University & Shandong academy of medical sciences, Tai’an, 271016 China; 4 Department of Infectious Disease, Tai’an Central Hospital, Tai’an, 271000 China

**Keywords:** Infectious diseases, Preventive medicine

## Abstract

Shandong Province is an area of China with a high incidence of haemorrhagic fever with renal syndrome (HFRS); however, the general epidemic trend of HFRS in Shandong remains unclear. Therefore, we established a mathematical model to predict the incidence trend of HFRS and used Joinpoint regression analysis, a generalised additive model (GAM), and other methods to evaluate the data. Incidence data from the first half of 2018 were included in a range predicted by a modified sum autoregressive integrated moving average-support vector machine (ARIMA-SVM) combination model. The highest incidence of HFRS occurred in October and November, and the annual mortality rate decreased by 7.3% (p < 0.05) from 2004 to 2017. In cold months, the incidence of HFRS increased by 4%, −1%, and 0.8% for every unit increase in temperature, relative humidity, and rainfall, respectively; in warm months, this incidence changed by 2%, −3%, and 0% respectively. Overall, HFRS incidence and mortality in Shandong showed a downward trend over the past 10 years. In both cold and warm months, the effects of temperature, relative humidity, and rainfall on HFRS incidence varied. A modified ARIMA-SVM combination model could effectively predict the occurrence of HFRS.

## Introduction

Haemorrhagic fever with renal syndrome (HFRS) is primarily distributed throughout Eurasia. However, China has one of the highest incidence rates of HFRS and is the location for the most serious epidemics. Furthermore, China has the highest number of patients with HFRS in the world^1,2^, and Shandong Province is a focal point for HFRS incidence within the country. Different regions have evolved different hantavirus subtypes. In China, HFRS is mainly caused by the Hantaan and Seoul viruses^3^. Hantavirus was first successfully isolated in 1978^4^, and currently, more than 21 types are known to cause human diseases^5^. The incubation period of HFRS is typically 2–3 weeks. Clinical manifestations include fever, bleeding, kidney damage, and hypotension depending on the type of hantavirus^6^. In the Americas, infection can lead to hantavirus pulmonary syndrome, while in Europe and Asia, it may lead to HFRS^7^. HFRS is also a risk factor for venous thromboembolism, myocardial infarction, and stroke^8^. Some strains may result in high case fatality rates^9^.

Rodents are natural hosts and the main source of human infection. Hantavirus-positive rates vary among rodent species, with *Apodemus agrarius*, *Rattus norvegicus*, *R*. *flavipectus*, *Cansumys canus*, and *Mus musculus* having positive rates of 6.8%, 0.5%, 0.2%, 0.3%, and 0.2%, respectively^4^. In Zibo, a prefecture-level city in Shandong Province, hantavirus antigens were detected in 5.2% of rodents, and in residential areas, the brown rat (*R*. *norvegicus*) and the house mouse (*M*. *musculus*) had infection rates of 5.3% and 2.4%, respectively^10^. One report indicated that *M*. *musculus*, a non-traditional animal host of hantavirus, may play an important role in hantavirus transmission in Qingdao City in Shandong Province^3^. Risk factors for hantavirus infection include involvement in outdoor activities, such as working in fields and forests, and exposure to peridomestic rodents. High rodent population densities also likely contribute to HFRS outbreaks^11^.

HFRS is transmitted to humans chiefly through rodents, and this transmission can be affected by meteorological factors^12^. Several studies have explored the association between meteorological variables (especially temperature) and HFRS^12,13^. However, the complex relationship between climate and hantavirus infections needs further research^14^. In this study, we investigated other important factors affecting the incidence of HFRS. An epidemiological analysis was conducted on the epidemic characteristics of HFRS in Shandong Province, with an aim to provide a theoretical basis for formulating reasonable disease prevention and control measures. Our study had the following characteristics: (1) a large number of cases, (2) a long time span, and (3) an increased predictive accuracy owing to twice revising a time series model.

## Results

### Comparison of HFRS values between shandong and other provinces

From 2004 to 2017, there were 20,981 cases of HFRS and 247 deaths in Shandong Province, which accounted for 11.7% and 14.1% of the national total, respectively (Shandong accounts for approximately 7.1% of the national population). From 2004 to 2017, the average annual incidence and mortality rates of HFRS in Shandong Province were 15.30/100,000 and 0.18/100,000, respectively, both of which exceeded the national average (p < 0.05).

As shown in Supplementary Figs S1 and S2, the incidence and number of deaths due to HFRS in Shandong Province were among the highest in China, indicating that the region is highly endemic.

### Seasonal comparisons

**Figure 1:**
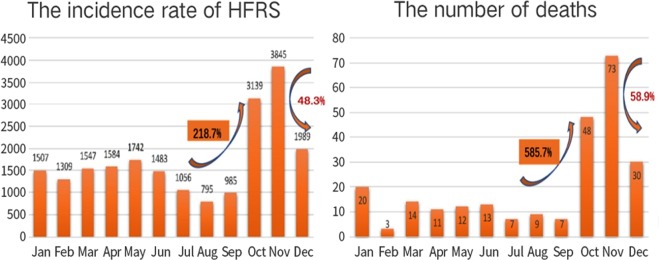
Monthly distribution of HFRS cases in Shandong from 2004 to 2017. The number of cases and deaths experienced the highest percent increase from September to October at 218.7% and 585.7%, respectively. The number of cases and deaths in the period from November to December showed the largest and second largest declines at 48.3% and 58.9%, respectively.

The total number of HFRS cases per month in Shandong Province from 2004 to 2017 is shown in Fig. 1. Peak onset occurred in October, November, and December of each year. The total number of cases during these months was 8,973, and the total number of deaths was 151, which accounted for 42.8% and 61.1% of the total number of cases over 14 years, respectively. Furthermore, Fig. 1 shows that the number of cases increased from 985 in September to 3139 in October, accounting for the highest percent increase in the number of cases at 218.7%. Likewise, fatality rose from 7 in September to 48 in October, a 585.7% increase. The highest percent decrease in the number of cases was 48.3% (from 3845 in November to 1989 in December), and for deaths, the percentage was 58.9% (fatality decreased from 73 in November to 30 in December). Morbidity and mortality per month are shown in Supplementary Figs S3 and S4. From 2004 to 2006, the incidence peaked during the winter months and from February to May.

**Figure 2:**
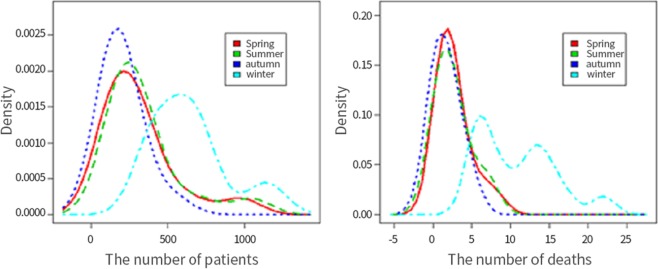
Density curves showing the number of patients and deaths per season from 2004 to 2017. The test of equal densities between winter and the other three seasons was p < 0.05 for all.

**Figure 3:**
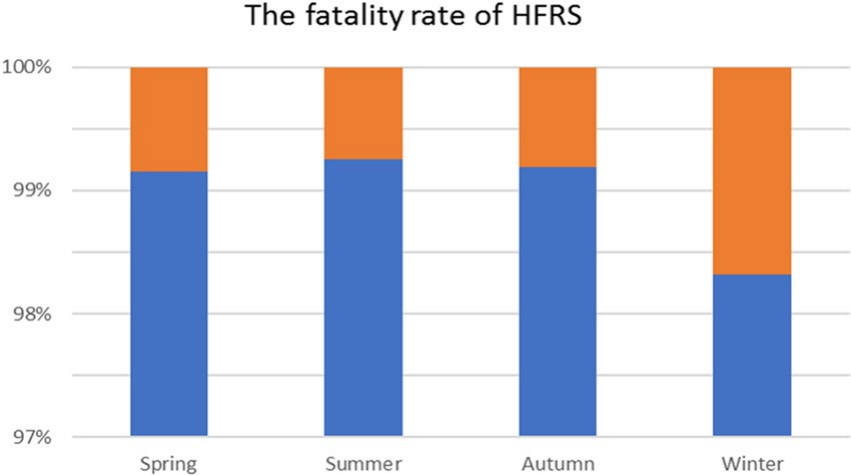
The fatality rate of HFRS in four different seasons. From 2004 to 2017, the fatality rate of HFRS in winter was significantly higher than in other seasons (p < 0.05).

Figure 2 shows a density curve for the number of patients and deaths per season from 2004 to 2017. Density maps for spring, summer, and autumn showed equal densities (p > 0.05), but these differed significantly from the density map for winter (p < 0.05). Figure 3 shows that the case fatality rate for winter (October, November, and December) was 1.68% higher than that for other seasons (spring 0.85%; summer 0.75%; and autumn 0.81%; p < 0.05).

### Overall incidence, mortality, and fatality trends

Overall, the incidence of HFRS decreased over the years examined (Fig. 4.1). The HFRS incidence was 4.02 cases per 100,000 in 2004 and 1.27 cases per 100,000 in 2017. From 2004 to 2007, the incidence rate decreased by 38.0% annually (p < 0.05). However, from 2007 to 2013, the rate increased by 9.5% (p > 0.05) and then decreased by 10.2% per year (p > 0.05) from 2013 to 2017. Moreover, HFRS-associated mortality decreased (Fig. 4.2). The mortality rate was 0.04 cases per 100,000 in 2004 and 0.01 cases per 100,000 in 2017. From 2004 to 2017, the annual mortality rate decreased by 7.3% (p < 0.05).

**Figure 4:**
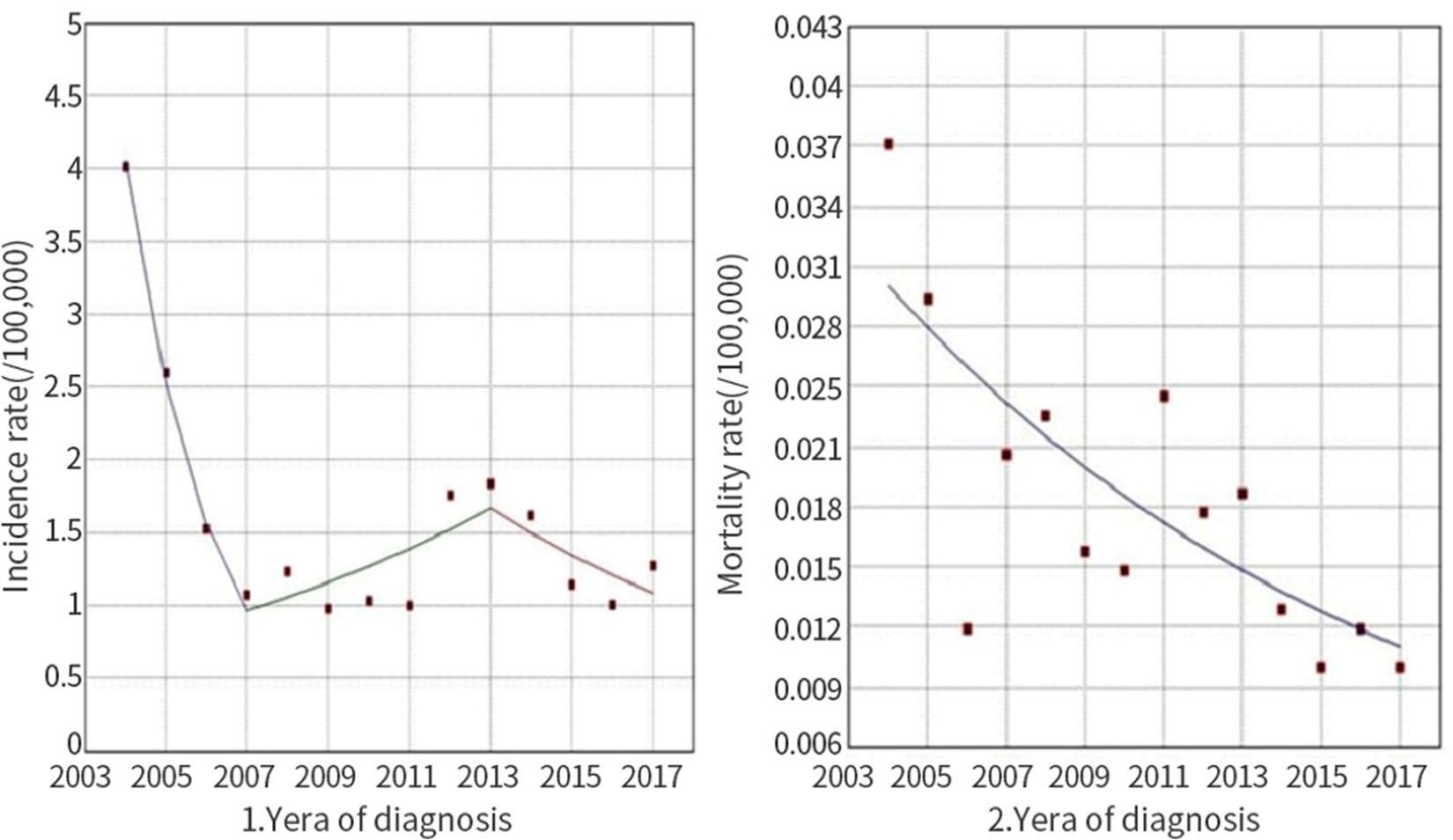
Trends in HFRS incidence and mortality rates from 2004 to 2017. Trend for the incidence rate of HFRS from 2004 to 2017 as calculated by Joinpoint regression analysis. (1) The annual incidence rate decreased by 38% every year from 2004 to 2007 (p < 0.05). From 2007 to 2013, the annual incidence increased by 9.5% (p > 0.05). From 2013 to 2017, the incidence rate decreased by 10.2% per year (p > 0.05). (2) From 2004 to 2017, the annual mortality rate decreased by 7.3% (p < 0.05).

Trends in the HFRS case fatality rate from 2004 to 2017 were calculated using Joinpoint regression (Supplementary Fig. S5), and the changes were not obvious. As shown in Fig. S5, the annual case fatality rate increased by 21.9% from 2004 to 2008 (p > 0.05) but decreased by 9.6% from 2008 to 2017 (p > 0.05).

### Correlation and generalised additive model

A Spearman correlation analysis showed that air temperature, relative humidity, and precipitation were negatively correlated with the monthly incidence of HFRS for each year (p < 0.05). There was no correlation between sunshine duration or evaporation and the incidence of HFRS. Pearson correlation analysis also showed no significant correlation between annual GDP per capita and the incidence of HFRS (p = 0.099, Fig. 5.1). However, annual GDP per capita and mortality were significantly positively correlated (p < 0.05, Fig. 5.2).

**Figure 5:**
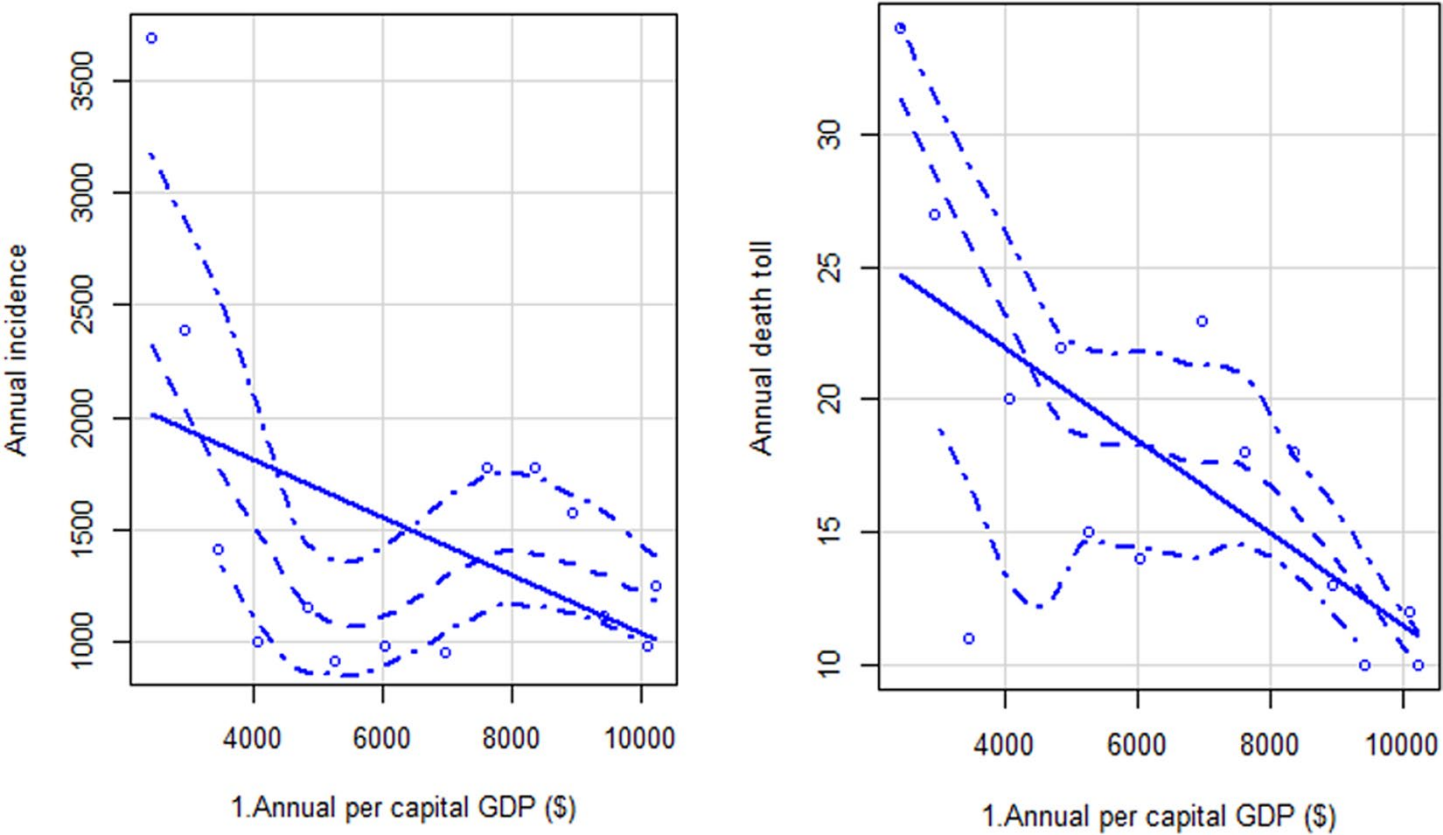
Scatter plot of annual gross domestic product (GDP) per capita and incidence (1) and mortality rate (2) of HFRS.

An unsupervised cluster analysis showed that the months of the year clustered into four groups: October and November; December, January and February; March, April and May, and; June, July, August, and September (Supplementary Fig. S6).

The estimated results of the basic model parameters from the generalised additional model (GAM) are shown in Supplementary Tables S2 and S3.

**Figure 6:**
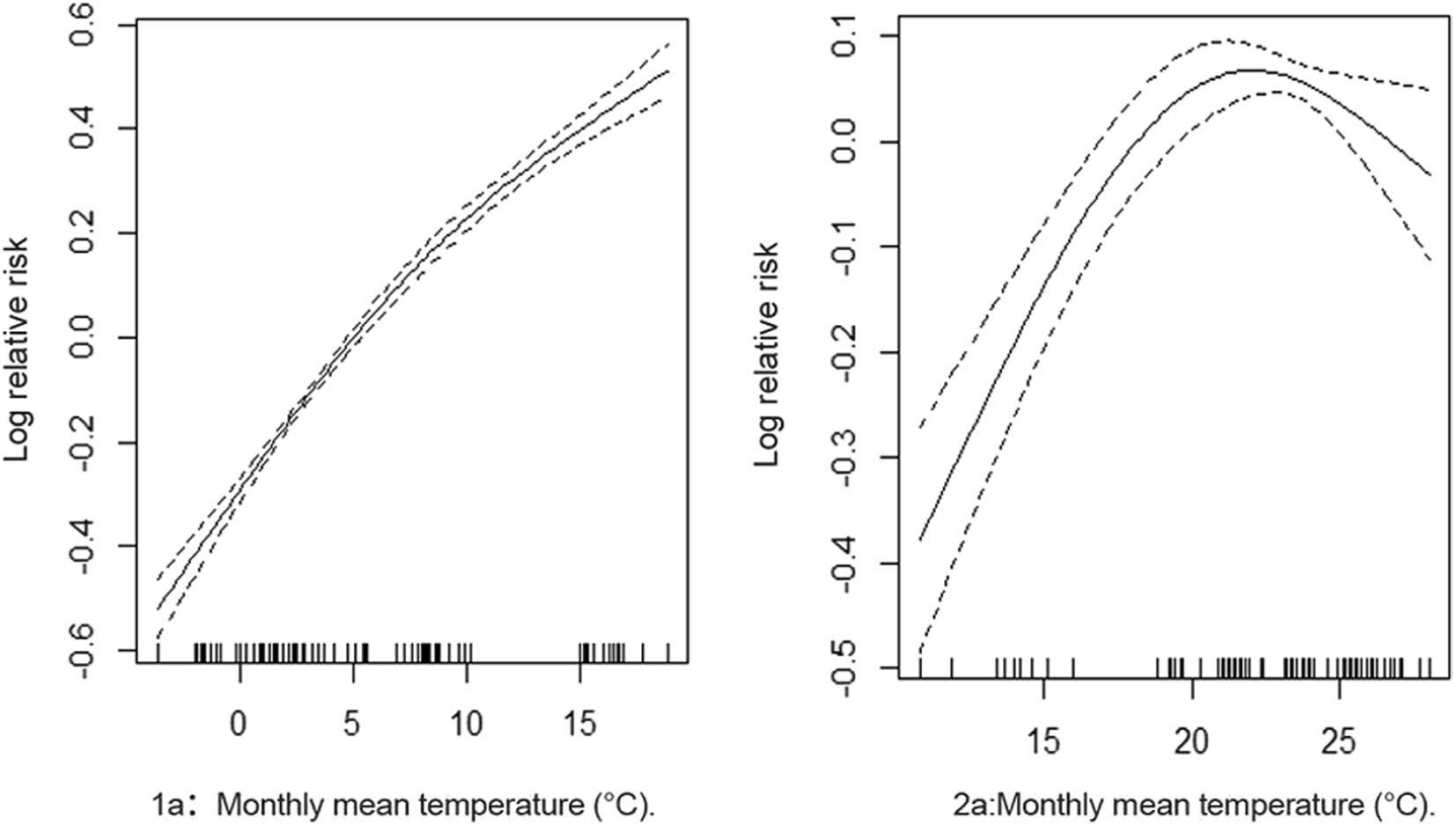
The relationship between monthly mean temperature and the incidence of HFRS (1**a** cold months; 2**a**) warm months).

In both cold and warm months, temperature had an effect on HFRS. In cold months, for every 1 °C increase in average temperature, the incidence of HFRS increased by 4% (Fig. 6:1a). In warm months, for every 1 °C increase in average temperature, the incidence of HFRS increased by 2% (Fig. 6:2a).

Relative humidity had a negative effect on the pathogenesis of HFRS. In cold months, for every 1% increase in relative humidity, the incidence of HFRS decreased by 1% (Supplementary Fig. S7:1b). However, in warm months, every 1% increase in relative humidity saw the incidence of HFRS decrease by 3% (Supplementary Fig. S7:2b).

In cold months, rainfall also had an effect on the incidence of haemorrhagic fever (p < 0.01, Supplementary Table S2). For every 1-mm increase in rainfall, the incidence of HFRS increased by 0.8% (Supplementary Fig S8:1c). However, during the warm months, rainfall had no effect on the incidence of HFRS (p > 0.05, Supplementary Table S3; Fig. S8:2c).

### Time series analysis

**Figure 7:**
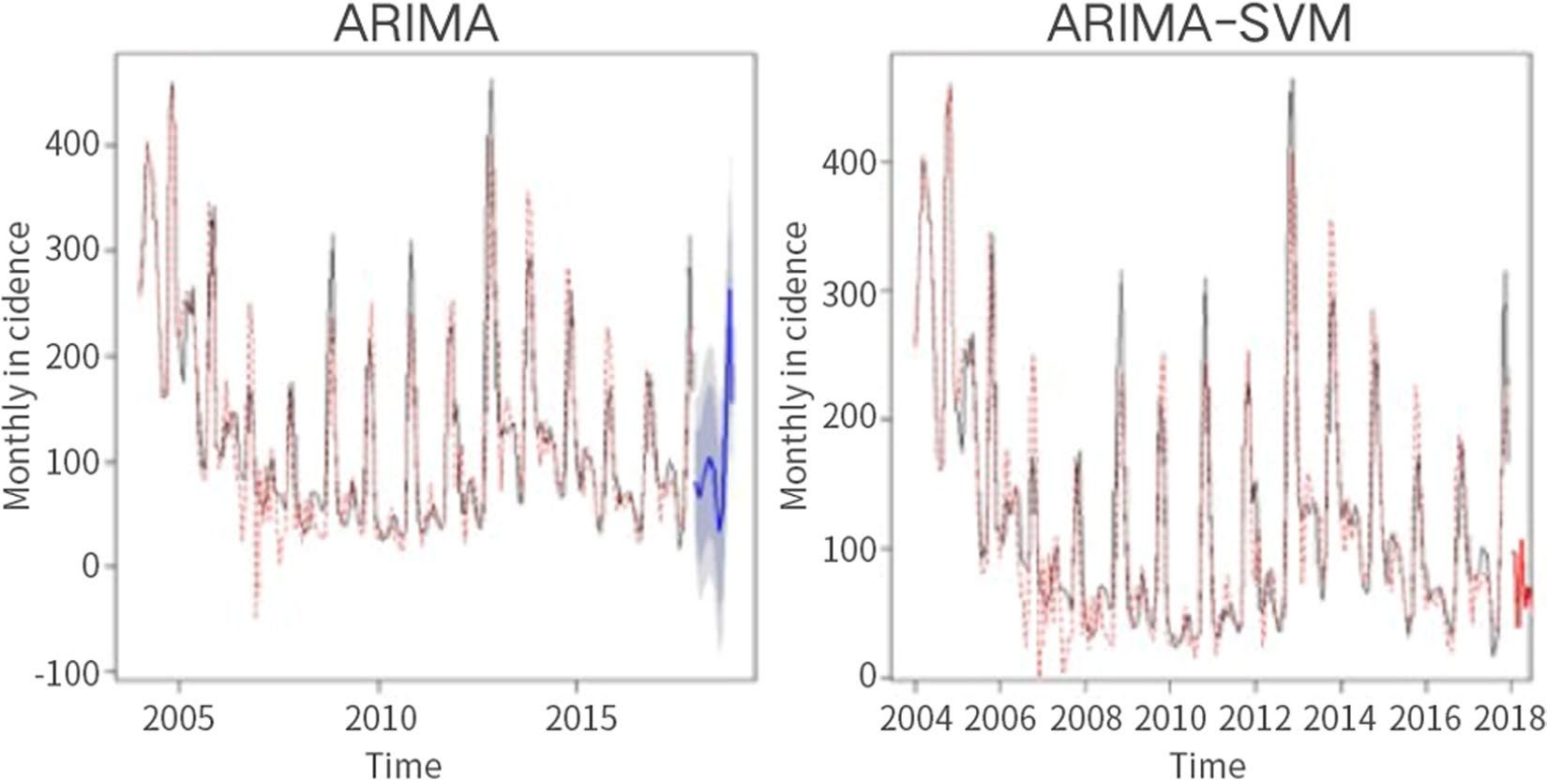
Autoregressive integrated moving average (ARIMA) and ARIMA-support vector machine (SVM) models and their predictions.

Because there was no obvious trend in the time series diagram, it was difficult to ascertain whether the time series was stable. Stationary series typically have short-term correlations, and the autocorrelation coefficient quickly decays to zero. Our autocorrelation-coefficient diagram did not show this trend; therefore, we assumed that the time series was not stationary and needed to be differentiated (Supplementary Fig. S9). After differentiating, the time series always randomly fluctuated near a constant value. Furthermore, the fluctuation range was bounded; thus, we assumed that the time series after differentiating was stable (Supplementary Fig. S10). Using the automatic order function in the R programming language (R Foundation for Statistical Computing, Vienna, Austria), we obtained the ARIMA (2, 1, 1) (0, 1, 1) [12] model. After passing a white noise test, the model was used for forecasting, and we obtained the predicted values of the ARIMA model. These were then added to the predicted value of the SVM model to obtain the predicted value of the combination model (Fig. 7). By subtracting the residual correction value from the predicted value of the combination model, we attained the final, modified prediction value of the combination model.

We tested the validity of this model based on 95% confidence intervals. These refer to the estimated intervals of the total parameters constructed by sample statistics (Table 1). The confidence interval of a probabilistic sample is an interval estimate of a population parameter of that sample.

**Table 1:** A comparison of the modified ARIMA-SVM combination model forecast and network report data.

Month (2018)	Number of cases	ARIMA-SVM	95% CI, lower limit	95% CI, upper limit
January	83	97	21	172
February	47	96	0	196
March	78	39	0	145
April	67	108	0	216
May	75	55	0	165
June	77	70	0	183

Abbreviations: ARIMA-SVM, autoregressive integrated moving average-support vector machine; CI, confidence interval.

## Discussion

This study analysed HFRS characteristics in Shandong, an area with a high incidence of the disease. A study of 20 patients showed that the incubation period for hantavirus infection ranged from 7 to 39 days^15^. The average incubation period of hantavirus-induced cardiopulmonary syndrome was reported to be 18.5 days^16^. However, because of the immune response, HFRS virus infection does not necessarily translate into illness. In this study, we considered only climatic factors and time points at which patients developed the disease. We did not investigate when they were infected with hantavirus because many people did not exhibit symptoms, even when infected.

Numerous reports have indicated that HFRS typically affects male farmers (over 60%) who live in rural areas. In terms of age distribution, patients are mostly youths and middle-aged adults^10,17^. Zheng *et al*. reported that farmers accounted for approximately 84% of HFRS patients in Shandong; more than 70% of patients were men, and approximately 80% were aged between 30 and 70 years^18^. The ratio of men to women is reported to be 3:1 in Shaanxi Province, China^7^. However, case fatality rates are higher among women than among men^19^. Cultivating land is considered to be the highest risk factor for HFRS^20^, and these data show that HFRS patients in China are mainly farmers.

One report indicated that HFRS cases peak concurrently with two important annual agricultural events, the spring and autumn harvests^4^. In Shandong Province, the autumn harvest occurs every year in late September, while the incidence of HFRS sharply increases in October, peaks in November, and then displays a substantial drop in December. The autumn harvest and its related work lasts for approximately 1–2 months and coincides with the peak incidence of HFRS. Given that the incubation period of HFRS is approximately 2–3 weeks, we speculate that peak incidence is related to the late September autumn harvest in rural areas. We further speculate that the autumn harvest is a possible cause for the winter-onset peak, which leads to a large number of young and middle-aged farmers working on farms and coming into close contact with sources of HFRS infection, thus increasing their chances of becoming infected. In addition, rats and other animals carrying the HFRS virus lack wild sources of food during the autumn harvest, and a large number of rats may enter the farmers’ homes, leading to infection. These reasons may explain why HFRS patients in Shandong are chiefly young and middle-aged male farmers and why the number of HFRS patients suddenly increases in October and sharply decreases in December.

In Shandong, young and middle-aged individuals who participate in agricultural production are at a high risk of contracting HFRS. Therefore, these individuals should consider receiving the inactivated hantavirus vaccine. In endemic areas, the inactivated hantavirus vaccine may offer effective protection^4,21^. The Korean army has widely used this vaccine to prevent HFRS and has reduced the incidence of the disease in this population^22^.

Overall, the incidence of HFRS decreased over the years we investigated. This decrease may be attributed to economic development, improved sanitation, and the use of vaccines. However, there have been no substantial changes in the incidence since 2007. In the future, precautions should be taken by high-risk groups during the autumn harvest season to reduce the incidence of HFRS.

Many studies have reported correlations between meteorological factors and HFRS. The number of monthly HFRS cases decreased by 5.5% when the temperature increased by 1 °C. Moreover, the number of incident cases decreased by 0.075% when the aggregate rainfall increased by 1 mm^13^. In addition to seasonal factors, HFRS incidence may be correlated to rodent density, with increases in rodent density potentially leading to outbreaks of HFRS^23^. HFRS cases were also associated with rainfall with a 3-month lag time and temperature with a 4-month lag time^23^. The number of HFRS epidemics also increased after rainy seasons^4^. In contrast, a negative correlation was observed between the incidence of HFRS and summer temperatures^24^. In our study, a correlation analysis revealed that the incidence of HFRS was negatively correlated with temperature, relative humidity, and precipitation. Our findings are similar to those of other studies^13,24^. As meteorological factors and HFRS displayed a time series when we performed a correlation analysis between them, a Pearson analysis was performed, where pseudo-regression tended to occur. Therefore, using the year as a unit, the monthly incidence was divided into three categories (low, moderate, and high), and Spearman analysis was performed. We used the GAM to further understand the relationship between meteorological factors and the incidence of HFRS. The months of the year were divided into two groups according to the differences in meteorological factors. The temperature was higher during April to September (group 2) than during the other months; all of the other months were placed in the lower temperature group 1. Our grouping was different from that used by other researchers^4,13,23^ and as we believe, more accurate. In the GAM, we found that in cold months, for every 1 °C increase in temperature, the incidence of HFRS increased by 4%, the relative humidity increased by 1%, the incidence of HFRS increased by 1%, the rainfall increased by 1%, and the incidence increased by 0.8%. However, in warmer months, for every 1 °C increase in temperature, the incidence of HFRS increased by 2%, the relative humidity increased by 1%, and the incidence increased by 3%. Rainfall had no effect on the incidence. This result is somewhat different from that of the Spearman correlation analysis mentioned previously, probably because that analysis used the year as a whole. As the incidence of HFRS is concentrated in winter, the Spearman correlation analysis found a negative correlation between temperature and incidence. However, in the GAM subgroup analysis, temperature increased during the cold season and was accompanied by an increase in incidence. This conclusion does not, however, conflict with that of the Spearman correlation analysis. During the cold season, the highest incidence of HFRS occurred in October and November, while the lowest temperatures occurred in December and January. From November to December, the temperature decreased and the incidence decreased, and the subgroup analysis showed a positive effect on temperature and incidence. The Spearman correlation analysis is from the overall analysis, while the GAM is from the local analysis; and hence, the latter analysis is more detailed.

HFRS has a negative relationship with GDP and is significantly associated with croplands, sows, and population density^17^. The urban population density of Shandong is much higher than that of rural areas. However, the incidence of HFRS in Shandong is primarily observed in the rural population; thus, the population density did not have a substantial impact on HFRS incidence. The development of the social economy has greatly influenced the spread of hantavirus^25^. HFRS incidence and urbanisation were strongly positively correlated from 1963 to 1990 but were negatively correlated from 1991 to 2010^26^. Urbanisation may have promoted the incidence of HFRS owing to poor sanitary and economic conditions. In our research, the association between annual GDP per capita and mortality was statistically significant (p < 0.05). Recently, improvements in health conditions have reduced the incidence of HFRS. Urbanisation and agricultural modernisation have reduced both the intensity and time farmers labour in the fields, which may have reduced the incidence of HFRS. Overall, the development of the economy seems to have helped prevent and control HFRS.

The multi-host ecology of zoonoses leads to complex dynamics, and analysis tools such as mathematical modelling are essential to develop effective prevention and control strategies^27^. Many scholars have used different methods to establish mathematical models to predict HFRS incident trends^12,13,28,29^. These models generally include meteorological factors, such as temperature, humidity, and rainfall, and geographical factors, such as land type. Considering that the incidence of diseases in Shandong differs from that in other areas, it is necessary to build a model specific for this region to predict the incidence of diseases. In this study, we used long-term and short-term memory models and a neural network model to predict haemorrhagic fever, but the residuals were too large to be used. The time series prediction method in the ARIMA model can also predict the linear part of the sequence quite well, but it cannot analyse the nonlinear parts. However, the SVM model in a machine learning algorithm is suitable for solving nonlinear problems and working with small sample sizes. Furthermore, the data requirements are not high, and a global optimal solution can be found. However, because a single mathematical model may not be very accurate, we used an ARIMA-SVM combination forecasting model to improve predictive accuracy. We also corrected the ARIMA-SVM combination model and a meaningful predicted value of the combined model could be found. The modified ARIMA-SVM combination prediction model is an effective method for predicting the incidence of HFRS.

This study has some limitations. First, some patient data, including patient age, occupation, and specific place of residence, were missing. Second, for further HFRS virus and subtype detection, it is necessary to capture mice from high-incidence areas, as their density distribution across different seasons may also correlate with disease occurrence. Third, it was difficult to determine the density distribution of HFRS patients over a wide range of times. Recording detailed patient personal data during hospital stays would be beneficial for future studies.

## Conclusion

From 2004 to 2017, the number of HFRS cases and deaths in Shandong showed overall downward trends. Each year in Shandong Province, the highest incidence of HFRS occurs in October and November after the autumn harvest. Using the GAM model, our analysis found that in cold months, for every unit increase in temperature, relative humidity, and rainfall, the incidence of HFRS increased by 4%, −1%, and 0.8% respectively, and in warm months, the incidence increased by 2–3%, and 0% respectively. A modified ARIMA-SVM combination model could effectively predict the occurrence of HFRS.

## Methods

### Data

Patient data were obtained from the Public Health Science Data Center of the Chinese Center for Disease Control and Prevention (http://www.chinacdc.cn/) and Shandong Province infectious diseases notification data (http://www.sdcdc.cn/index.html). We extracted HFRS incidence and mortality data by month in Shandong from 2004 to 2017. Meteorological data were obtained from the Shandong Meteorological Bureau. We used the meteorological data from several regions to calculate an average value for the entire province (Supplementary Table S1). The per capita gross domestic product (GDP) of Shandong Province was obtained from the National Bureau of Statistics (http://www.stats.gov.cn/english/). The exchange rate from RMB (¥) to U.S. dollars ($) was calculated as 6.8:1.

Shandong Province is located on the east coast of China, covers an area of 156,700 square kilometres, and has a population of approximately 98 million (2014). The land area (north latitudes (34°22′52″ to 38° 15′02″, 53″ to 122°) and east longitude 114°19′) is characterised as a warm temperate zone with a monsoon climate (Supplementary Fig. S11). The inland plains account for 55% of the total land area, whereas the mountainous regions, hilly areas, and other land account for 15.5%, 13.2%, and 16.3% of the total land area, respectively.

### Ethical considerations

We received approval from the ethical committee of Shandong First Medical University for conducting this research (ethics approval No: 2019101). All analysed data were anonymised. Shandong First Medical University waived the need for patients to sign informed consent for the use of research data.

### Methods

Density estimates, along with the incidence and mortality rates, were calculated for the four seasons of spring, summer, autumn, and winter, and density differences were examined. Joinpoint regression analysis was used to evaluate the incidence, mortality, and case fatality rates of HFRS in Shandong Province from 2004 to 2017. We analysed the correlations between HFRS and meteorological factors, as well as per capita GDP. In these correlation analyses, different grouping forms were adopted, which could effectively avoid pseudo-regression. Time series analysis methods were used to predict future epidemics. We predicted HFRS trends by calibrating an autoregressive integrated moving average-support vector machine (ARIMA-SVM) combination model.

### Statistical analysis

#### Basic characteristics

We used a general description of the affected population. ArcGIS 10.2 (ESRI, Redland, CA, USA) was used for mapping.

#### Joinpoint regression analysis

A Joinpoint regression model was established for the incidence of HFRS in Shandong Province from 2004 to 2017 using the Joinpoint (https://surveillance.cancer.gov/joinpoint/) statistical software for the analysis. Joinpoint regression analysis was used to observe the annual percent change in incidence, mortality, and case fatality rates of HFRS. The model uses a piecewise linear regression approach to determine whether rates over time (2004–2017) are best described by a straight line or by multiple linear segments^30^.

#### Correlation analysis and GAM

We categorised the months of the year according to the number of annual HFRS cases as high-incidence, moderate-incidence, and low-incidence months (4 months each per year) and analysed the Spearman correlations between the three categories and meteorological factors. The Pearson correlations between annual GDP and annual incidence and mortality were analysed. These analyses were implemented in the R programming language (https://www.r-project.org/).

GAM is a nonparametric extension of the traditional generalised linear model, which can effectively deal with complex nonlinear relationships between explanatory variables and effect variables. Many reports in the literature have described GAM in detail^31–33^. Currently, it has been widely used in modelling the effect of meteorological factors on population health events^34,35^. The basic model was established using the “gam” function in the “mgcv” package (https://cran.r-project.org/web/packages/mgcv/index.html). HFRS was Poisson distributed using fit < -gam(f~Date + a + b + c + s, family = Poisson, data = data) with a: monthly mean temperature, b: monthly relative humidity, c: monthly rainfall, f: monthly incidence, and s: cold or warm months. When s = 1, a relatively cold month is indicated, from October to December, and January to March. When s = 2, a relatively warm month is indicated, from April to September. The “Gamm” function was used for effect-dose analysis.

#### Time series analysis

The R programming language was adopted to model and analyse the data. We first tested the stationarity of the time series. If it was not stable, we carried out the difference. White noise was used to test the randomness of sequences. Through the white noise test, the ARIMA model was used for fitting and forecasting^36^. The predicted value of ARIMA was combined with the SVM residual value to obtain the predicted value of an ARIMA-SVM combination model. The error of the combination model was then corrected. We used the ARIMA- SVM model to forecast the incidence of disease from 2016 to 2017 and obtain the prediction error for those 24 months. The average prediction error of the ARIMA- SVM model over 24 months was chosen as the error correction value of the ARIMA-SVM combination model. We corrected the ARIMA-SVM combination model to obtain more accurate prediction results. A p-value < 0.05 was considered statistically significant.

## Supplementary information

Supplementary Information
