# Supplementary material for: Epidemiological and time series analysis of haemorrhagic fever with renal syndrome from 2004 to 2017 in Shandong Province, China
Source: Sci Rep. 2019 Oct 10;9:14644. doi: 10.1038/s41598-019-50878-7 (PMC6787217; doi:10.1038/s41598-019-50878-7)
Supplement: Supplementary file 1 — Supplementary Information [file 41598_2019_50878_MOESM1_ESM.docx]

**Supplementary Information**

**Epidemiological and time-series analysis of haemorrhagic fever with renal syndrome from 2004 to 2017 in Shandong Province, China**

Chao Zhang, Xiao Fu ,Yuanying Zhang, Cuifang Nie, Li Liu, Haijun Cao, Junmei Wang, Baojia Wang, Shuyiing Yi, Zhen Ye

Table S1. Basic information on hemorrhagic fever and meteorological data from 2004 to 2017 in Shandong province

| Date | s | a (°C) | b (%) | c (mm) | d (mm) | e (hours) | f | g |
| --- | --- | --- | --- | --- | --- | --- | --- | --- |
| January−2004 | 1 | -0.9 | 56.7 | 2.5 | 48.2 | 176.2 | 257 | 1 |
| February−2004 | 1 | 4.1 | 48 | 16.8 | 111.2 | 217.5 | 316 | 0 |
| March−2004 | 1 | 8.1 | 53 | 4.1 | 148.7 | 211.8 | 404 | 1 |
| April−2004 | 2 | 14.6 | 57 | 35.3 | 186.6 | 232.4 | 384 | 2 |
| may−2004 | 2 | 18.8 | 62.3 | 91.5 | 196 | 231.3 | 366 | 3 |
| June−2004 | 2 | 23.2 | 67 | 109.7 | 208.5 | 174.6 | 296 | 2 |
| July−2004 | 2 | 25.7 | 79 | 233.3 | 164.6 | 168.6 | 210 | 2 |
| August−2004 | 2 | 24.6 | 78.7 | 189.1 | 137.9 | 147 | 160 | 0 |
| September−2004 | 2 | 21.8 | 68.7 | 63.4 | 148.1 | 206.6 | 164 | 1 |
| October−2004 | 1 | 15.3 | 57.7 | 10 | 143.1 | 211.6 | 432 | 8 |
| November−2004 | 1 | 8.8 | 63.7 | 51.1 | 93.1 | 182.6 | 461 | 12 |
| December−2004 | 1 | 2.1 | 67.3 | 9.4 | 50.7 | 126.2 | 233 | 2 |
| January−2005 | 1 | -1.7 | 52 | 0.1 | 53.6 | 209.9 | 210 | 4 |
| February−2005 | 1 | -1.6 | 60.7 | 25.3 | 48 | 144.8 | 175 | 1 |
| March−2005 | 1 | 5.5 | 50.3 | 3 | 149.5 | 238.5 | 254 | 3 |
| April−2005 | 2 | 15.1 | 49.7 | 25.1 | 225.4 | 265.8 | 240 | 1 |
| May−2005 | 2 | 18.8 | 55.7 | 58.5 | 250.6 | 293.4 | 266 | 0 |
| June−2005 | 2 | 25.4 | 60 | 108.3 | 276.7 | 246.8 | 194 | 3 |
| July−2005 | 2 | 26.5 | 76.3 | 144.6 | 186 | 176.9 | 135 | 1 |
| August−2005 | 2 | 24.9 | 78.7 | 177.6 | 164.2 | 192.7 | 92 | 2 |
| September−2005 | 2 | 21 | 72.7 | 228.2 | 134.1 | 155.7 | 104 | 1 |
| October−2005 | 1 | 15.4 | 59 | 23.2 | 136.3 | 211.7 | 263 | 1 |
| November−2005 | 1 | 10.2 | 56.3 | 5.9 | 89.5 | 191.9 | 343 | 7 |
| December−2005 | 1 | -0.9 | 51 | 4.5 | 55.1 | 185.6 | 114 | 3 |
| January−2006 | 1 | 0 | 68.3 | 5.7 | 36.6 | 110.5 | 112 | 1 |
| February−2006 | 1 | 0.9 | 61 | 6.9 | 61.8 | 159.2 | 104 | 0 |
| March−2006 | 1 | 7.8 | 47.3 | 1.4 | 158.4 | 236.1 | 139 | 2 |
| April−2006 | 2 | 13.7 | 55.3 | 21.4 | 186.8 | 236.3 | 125 | 1 |
| May−2006 | 2 | 19.2 | 63 | 100.5 | 220 | 240.5 | 148 | 0 |
| June−2006 | 2 | 24.1 | 67.7 | 66.6 | 263.2 | 222.5 | 144 | 1 |
| July−2006 | 2 | 26 | 80 | 76.1 | 199.8 | 154.9 | 92 | 0 |
| August−2006 | 2 | 26.1 | 81.7 | 163.8 | 191.3 | 202.1 | 85 | 0 |
| September−2006 | 2 | 21.2 | 71.3 | 12.4 | 158.5 | 187.6 | 82 | 0 |
| October−2006 | 1 | 18.9 | 69 | 1 | 157.3 | 190.5 | 171 | 2 |
| November−2006 | 1 | 9.9 | 64.7 | 16.4 | 94.8 | 140.3 | 138 | 4 |
| December−2006 | 1 | 1.4 | 67.3 | 10.6 | 47.7 | 138.5 | 71 | 0 |
| January−2007 | 1 | -0.2 | 63 | 5.2 | 49 | 161 | 65 | 2 |
| February−2007 | 1 | 5.4 | 61.7 | 7.4 | 86.2 | 172.6 | 56 | 1 |
| March−2007 | 1 | 7.6 | 66 | 51.7 | 106.5 | 168.9 | 60 | 3 |
| April−2007 | 2 | 14 | 57.3 | 17.2 | 190.9 | 225.5 | 89 | 4 |
| May−2007 | 2 | 20.9 | 59.7 | 64.3 | 253.4 | 246.8 | 103 | 2 |
| June−2007 | 2 | 23.7 | 72.7 | 104.3 | 233.5 | 174.5 | 68 | 1 |
| July−2007 | 2 | 25.4 | 80.3 | 181.9 | 195.7 | 155.1 | 69 | 0 |
| August−2007 | 2 | 25.5 | 85.3 | 335.6 | 156.8 | 140.5 | 63 | 2 |
| September−2007 | 2 | 21.9 | 77.3 | 128.5 | 151.7 | 152.9 | 52 | 0 |
| October−2007 | 1 | 15 | 70 | 27.6 | 115.8 | 134.1 | 125 | 2 |
| November−2007 | 1 | 8.2 | 59.3 | 0 | 87.3 | 183.4 | 175 | 0 |
| December−2007 | 1 | 2.4 | 67 | 21.6 | 46.7 | 126.6 | 77 | 3 |
| January−2008 | 1 | -1.9 | 62.3 | 7.4 | 43.5 | 110.1 | 48 | 0 |
| February−2008 | 1 | 0.2 | 51 | 5.2 | 67.5 | 195.3 | 38 | 0 |
| March−2008 | 1 | 8.4 | 53 | 13 | 143 | 229.4 | 32 | 1 |
| April−2008 | 2 | 14 | 60.7 | 60.8 | 160.7 | 207.6 | 40 | 0 |
| May−2008 | 2 | 19.3 | 65.7 | 79.3 | 196.1 | 223.5 | 70 | 1 |
| June−2008 | 2 | 22.3 | 74.7 | 33.5 | 202.9 | 149.3 | 71 | 1 |
| July−2008 | 2 | 25.3 | 85.7 | 286.5 | 140.5 | 117.7 | 64 | 1 |
| August−2008 | 2 | 25.3 | 79.3 | 188.6 | 163.9 | 174.6 | 53 | 2 |
| September−2008 | 2 | 21.5 | 72.3 | 52.5 | 143.9 | 180.2 | 58 | 2 |
| October−2008 | 1 | 16.7 | 63.3 | 31.2 | 125.7 | 182.2 | 231 | 4 |
| November−2008 | 1 | 8.6 | 54.7 | 11.7 | 93.7 | 171.5 | 315 | 8 |
| December−2008 | 1 | 1.9 | 50 | 3.3 | 72.4 | 164 | 134 | 2 |
| January−2009 | 1 | -0.9 | 49.7 | 0.4 | 63.1 | 182.8 | 55 | 3 |
| February−2009 | 1 | 3.7 | 65 | 11.1 | 71.4 | 103.6 | 41 | 0 |
| March−2009 | 1 | 7.2 | 52.7 | 28.7 | 153.8 | 207.1 | 39 | 0 |
| April−2009 | 2 | 14.2 | 54.3 | 47.1 | 181.5 | 240.2 | 48 | 1 |
| May−2009 | 2 | 20.3 | 54.7 | 78 | 223.1 | 240.7 | 79 | 2 |
| June−2009 | 2 | 25.2 | 58 | 60.3 | 270.9 | 256.8 | 60 | 1 |
| July−2009 | 2 | 25.3 | 76.3 | 198.2 | 171.4 | 191 | 41 | 1 |
| August−2009 | 2 | 25.2 | 77.7 | 109.3 | 166.2 | 194.3 | 39 | 1 |
| September−2009 | 2 | 21.4 | 72.3 | 20.6 | 139.5 | 168.8 | 70 | 0 |
| October−2009 | 1 | 17.8 | 54.7 | 23.3 | 154.9 | 203.5 | 214 | 4 |
| November−2009 | 1 | 5.1 | 63 | 31.8 | 78 | 146.8 | 186 | 0 |
| December−2009 | 1 | 0.6 | 59 | 9.6 | 59.8 | 151.1 | 50 | 2 |
| January−2010 | 1 | -1.8 | 57.7 | 3.7 | 49.4 | 159.1 | 35 | 0 |
| February−2010 | 1 | 1.4 | 65 | 8.4 | 55.4 | 102.8 | 23 | 0 |
| March−2010 | 1 | 4.7 | 59.3 | 36 | 102.6 | 152.9 | 27 | 1 |
| April−2010 | 2 | 10.8 | 55.3 | 22.2 | 165.2 | 203.8 | 28 | 0 |
| May−2010 | 2 | 19.7 | 58.7 | 63.2 | 217 | 241.2 | 40 | 0 |
| June−2010 | 2 | 23.4 | 69.3 | 80 | 203.3 | 205.9 | 49 | 0 |
| July−2010 | 2 | 27.1 | 74.7 | 126.3 | 187.2 | 193.6 | 31 | 0 |
| August−2010 | 2 | 25.6 | 83 | 272.5 | 129.4 | 115.2 | 30 | 0 |
| September−2010 | 2 | 21.8 | 75 | 81 | 131.1 | 145.6 | 33 | 0 |
| October−2010 | 1 | 15.3 | 60.3 | 9.1 | 146.6 | 216.4 | 233 | 4 |
| November−2010 | 1 | 9.7 | 49.7 | 0 | 113.9 | 208.9 | 310 | 5 |
| December−2010 | 1 | 2.5 | 44 | 1.4 | 91 | 208 | 141 | 4 |
| January−2011 | 1 | -3.7 | 43 | 0.3 | 53.9 | 196.3 | 44 | 3 |
| February−2011 | 1 | 1.6 | 55.7 | 20.5 | 67.1 | 137.5 | 33 | 1 |
| March−2011 | 1 | 7.2 | 41.7 | 3.5 | 155.2 | 259.3 | 32 | 0 |
| April−2011 | 2 | 13.4 | 47.3 | 11.6 | 212.6 | 255 | 53 | 0 |
| May−2011 | 2 | 19.4 | 54 | 47.2 | 217.4 | 236.2 | 48 | 0 |
| June−2011 | 2 | 23.9 | 64 | 45.2 | 238.9 | 178.4 | 57 | 1 |
| July−2011 | 2 | 25.9 | 77.3 | 204.5 | 153.4 | 142.9 | 43 | 0 |
| August−2011 | 2 | 25.1 | 82 | 203.3 | 119.2 | 133.2 | 34 | 1 |
| September−2011 | 2 | 20.3 | 70 | 127.1 | 138.1 | 166.1 | 42 | 0 |
| October−2011 | 1 | 15.6 | 64 | 11.2 | 118.6 | 182 | 208 | 4 |
| November−2011 | 1 | 9.9 | 70.7 | 60.1 | 61.9 | 116.4 | 229 | 9 |
| December−2011 | 1 | 0.6 | 64 | 18.6 | 42.2 | 152.7 | 135 | 4 |
| January−2012 | 1 | -1.3 | 63 | 2.1 | 39.5 | 135.9 | 154 | 2 |
| February−2012 | 1 | 0.2 | 50 | 1.3 | 61.6 | 178.6 | 53 | 0 |
| March−2012 | 1 | 5.6 | 56.7 | 19.5 | 107.6 | 177.3 | 50 | 0 |
| April−2012 | 2 | 14.6 | 60.3 | 53.3 | 158.8 | 230.6 | 75 | 0 |
| May−2012 | 2 | 21.1 | 59.7 | 5.1 | 218.2 | 264.4 | 83 | 1 |
| June−2012 | 2 | 24 | 68 | 38.9 | 234.1 | 189.5 | 54 | 0 |
| July−2012 | 2 | 26.7 | 80 | 157.7 | 171.7 | 166 | 42 | 0 |
| August−2012 | 2 | 25.1 | 84.7 | 163.8 | 132.5 | 172.7 | 35 | 0 |
| September−2012 | 2 | 21.2 | 71 | 27.9 | 148.5 | 185.2 | 67 | 0 |
| October−2012 | 1 | 16.8 | 57.3 | 12.4 | 150.8 | 214.9 | 400 | 5 |
| November−2012 | 1 | 6.9 | 58.3 | 50.7 | 87.4 | 179.3 | 465 | 6 |
| December−2012 | 1 | -1.1 | 63.3 | 32.2 | 46.3 | 148.1 | 295 | 4 |
| January−2013 | 1 | -2 | 73.7 | 11.7 | 31.5 | 147.9 | 141 | 2 |
| February−2013 | 1 | 1 | 72.3 | 14.7 | 44 | 112.6 | 123 | 0 |
| March−2013 | 1 | 6.9 | 52.7 | 9.1 | 143 | 233.5 | 136 | 0 |
| April−2013 | 2 | 11.9 | 52 | 12.8 | 180.1 | 249.1 | 126 | 0 |
| May−2013 | 2 | 19.3 | 67 | 103.8 | 175.4 | 218.5 | 134 | 0 |
| June−2013 | 2 | 23.2 | 72 | 33.4 | 194.7 | 200.2 | 135 | 0 |
| July−2013 | 2 | 26.7 | 81.7 | 269.3 | 131 | 136.5 | 77 | 0 |
| August−2013 | 2 | 28 | 70.7 | 55.9 | 204.4 | 267.9 | 59 | 0 |
| September−2013 | 2 | 22.4 | 66 | 39.3 | 155.5 | 195.8 | 101 | 3 |
| October−2013 | 1 | 16.1 | 57.7 | 8 | 145.7 | 236.3 | 277 | 6 |
| November−2013 | 1 | 8 | 54.3 | 44.5 | 83.1 | 190.7 | 295 | 4 |
| December−2013 | 1 | 1.3 | 53 | 1.2 | 57.1 | 189.9 | 175 | 3 |
| January−2014 | 1 | 2.1 | 58.7 | 0.9 | 58.4 | 168.8 | 121 | 1 |
| February−2014 | 1 | 1.5 | 64 | 17.5 | 54.9 | 137.1 | 138 | 0 |
| March−2014 | 1 | 10.2 | 52.7 | 1.2 | 150.7 | 217.6 | 120 | 0 |
| April−2014 | 2 | 15.1 | 60.7 | 29.2 | 162.7 | 202.5 | 119 | 1 |
| May−2014 | 2 | 21.6 | 49 | 57.7 | 255.4 | 293.8 | 133 | 0 |
| June−2014 | 2 | 23.3 | 69.7 | 64.7 | 196.6 | 169.7 | 116 | 1 |
| July−2014 | 2 | 26.2 | 72 | 149.1 | 215.7 | 189.1 | 84 | 1 |
| August−2014 | 2 | 25.5 | 73 | 95.1 | 159.9 | 194.6 | 66 | 0 |
| September−2014 | 2 | 21.3 | 76 | 79.9 | 117.3 | 136.5 | 67 | 0 |
| October−2014 | 1 | 16.5 | 65.3 | 24.3 | 127.9 | 198.4 | 187 | 3 |
| November−2014 | 1 | 9.2 | 61 | 26 | 77.3 | 136.2 | 260 | 6 |
| December−2014 | 1 | 1.3 | 45.7 | 1.6 | 66.3 | 192.1 | 165 | 0 |
| January−2015 | 1 | 1.4 | 56.7 | 10.1 | 58.3 | 157.1 | 103 | 1 |
| February−2015 | 1 | 2.7 | 56.7 | 5.3 | 69.5 | 168.3 | 73 | 0 |
| March−2015 | 1 | 8.7 | 50.3 | 1.4 | 149.8 | 241.1 | 111 | 0 |
| April−2015 | 2 | 13.7 | 60 | 69.5 | 172 | 223.4 | 105 | 0 |
| May−2015 | 2 | 19.6 | 61 | 37.9 | 209.7 | 245.5 | 101 | 1 |
| June−2015 | 2 | 23.9 | 64 | 47.1 | 240.1 | 215.6 | 84 | 2 |
| July−2015 | 2 | 26.5 | 70.7 | 74 | 192.1 | 194.8 | 55 | 0 |
| August−2015 | 2 | 26.1 | 75 | 143.7 | 171.8 | 207.5 | 32 | 0 |
| September−2015 | 2 | 21.9 | 70.3 | 59.1 | 149.1 | 194.1 | 51 | 0 |
| October−2015 | 1 | 16.9 | 59 | 20.8 | 140.6 | 205.5 | 146 | 2 |
| November−2015 | 1 | 7.2 | 78.7 | 80 | 47.4 | 76.1 | 171 | 2 |
| December−2015 | 1 | 2.8 | 63.7 | 2.7 | 52 | 141 | 89 | 2 |
| January−2016 | 1 | -1.7 | 54.5 | 3 | 48.4 | 141 | 83 | 0 |
| February−2016 | 1 | 2.7 | 50.6 | 27.4 | 75.2 | 183 | 59 | 0 |
| March−2016 | 1 | 9.2 | 47.6 | 3 | 145.9 | 234 | 66 | 2 |
| April−2016 | 2 | 16 | 54.7 | 20.3 | 112.2 | 207 | 69 | 0 |
| May−2016 | 2 | 19.7 | 56.4 | 64.3 | 120.8 | 217.7 | 70 | 1 |
| June−2016 | 2 | 23.8 | 66.5 | 108 | 132.6 | 202.7 | 59 | 0 |
| July−2016 | 2 | 27 | 77.2 | 167.2 | 117.7 | 179 | 44 | 1 |
| August−2016 | 2 | 26.8 | 75 | 172.3 | 110.4 | 180.7 | 31 | 0 |
| September−2016 | 2 | 23.1 | 64.5 | 19.1 | 95.4 | 154.3 | 59 | 0 |
| October−2016 | 1 | 16.3 | 70.7 | 59.6 | 71.2 | 118 | 119 | 2 |
| November−2016 | 1 | 8.3 | 63.6 | 13.9 | 65.8 | 143.3 | 183 | 6 |
| December−2016 | 1 | 3.2 | 67.2 | 23.2 | 45.2 | 122 | 143 | 0 |
| January−2017 | 1 | 0.8 | 64.7 | 25.9 | 42.4 | 142.1 | 79 | 0 |
| February−2017 | 1 | 3.4 | 54 | 8.7 | 68.1 | 177 | 77 | 0 |
| March−2017 | 1 | 8.1 | 50 | 17 | 102.5 | 212.7 | 77 | 1 |
| April−2017 | 2 | 16 | 53 | 19.2 | 289.7 | 242.5 | 83 | 1 |
| May−2017 | 2 | 21.7 | 54.3 | 45.6 | 417.7 | 289.4 | 101 | 1 |
| June−2017 | 2 | 24.1 | 62.1 | 66.5 | 410 | 232.2 | 96 | 0 |
| July−2017 | 2 | 27.7 | 79 | 179.9 | 287 | 158.9 | 69 | 0 |
| August−2017 | 2 | 26.5 | 77.4 | 203.5 | 257.5 | 182.3 | 16 | 1 |
| September−2017 | 2 | 23.5 | 66.1 | 52.2 | 243.8 | 217.5 | 35 | 0 |
| October−2017 | 1 | 15.2 | 67.2 | 22.5 | 159.2 | 142.6 | 133 | 1 |
| November−2017 | 1 | 8.6 | 48.4 | 0.6 | 104.1 | 195.6 | 314 | 4 |
| December−2017 | 1 | 2.3 | 49.5 | 0.9 | 69 | 186.2 | 167 | 1 |

s: When s=1, a relatively cold month is indicated, from October to December, and January to March. When s=2, a relatively warm month is indicated, from April to September. a: Monthly mean temperature. b: Monthly relative humidity. c: Monthly rainfall. d: Monthly evaporation. e: Monthly sunshine duration. f: Monthly incidence. g: Monthly death toll.

Table S2 Parameter estimation for the GAM basic model in group 1.

|  | Estimate | Std.Error | Z | P |
| --- | --- | --- | --- | --- |
| (Intercept) | 5.7659548 | 0.0776428 | 74.26 | <0.01 |
| a | 0.0444164 | 0.001461 | 30.4 | <0.01 |
| b | -0.0145066 | 0.0013805 | -10.51 | <0.01 |
| c | 0.0080751 | 0.0006174 | 13.08 | <0.01 |
| Date | -0.0033836 | 0.0001809 | -18.7 | <0.01 |

Table S3 Parameter estimation for the GAM basic model in group 2.

|  | Estimate | Std.Error | Z | P |
| --- | --- | --- | --- | --- |
| (Intercept) | 6.86918 | 0.099575 | 68.985 | <0.01 |
| a | 0.02208 | 0.004067 | 5.428 | <0.01 |
| b | -0.0331 | 0.002083 | -15.883 | <0.01 |
| c | 0.00022 | 0.000256 | 0.86 | 0.39 |
| Date | -0.0084 | 0.000252 | -33.481 | <0.01 |


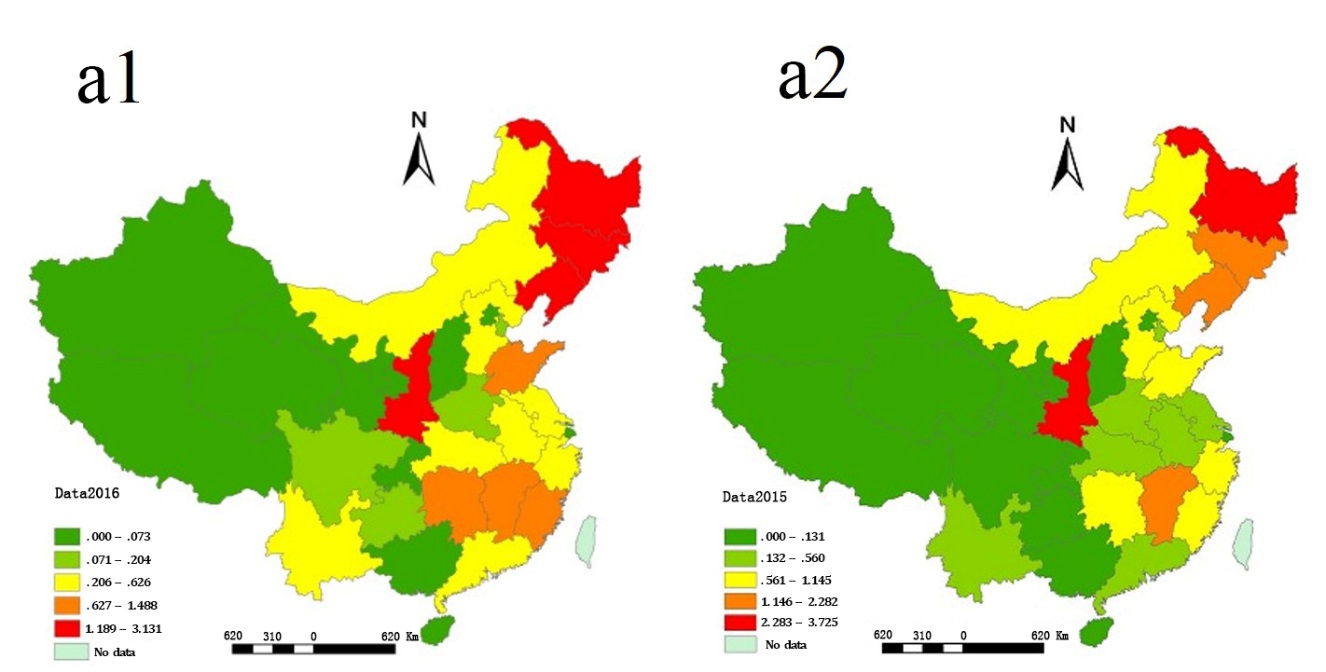


Figure S1-a1/ S1-a2. The incidence distribution of HFRS in China in 2016 (a1) and 2015 (a2). Darker colors indicate higher incidence. Created using ArcGIS 10.2.


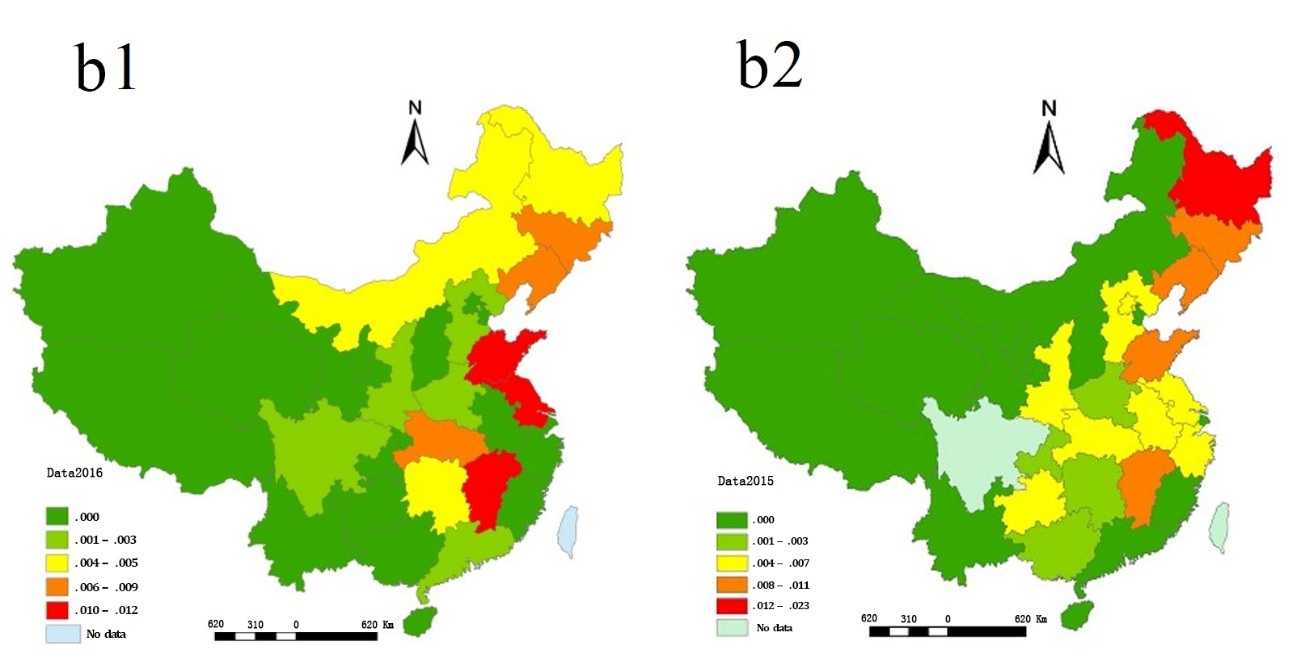


Figure S2-b1/ S2-b2. Distribution of the number of deaths from hemorrhagic fever in China in 2016 (b1) and 2015 (b2). Darker color indicates higher incidence.


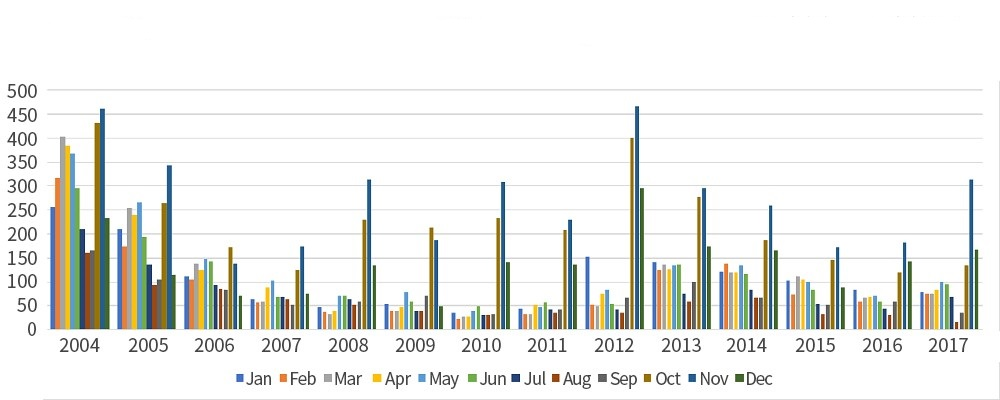


Figure S3. The number of hemorrhagic fever cases per month from 2004 to 2017.


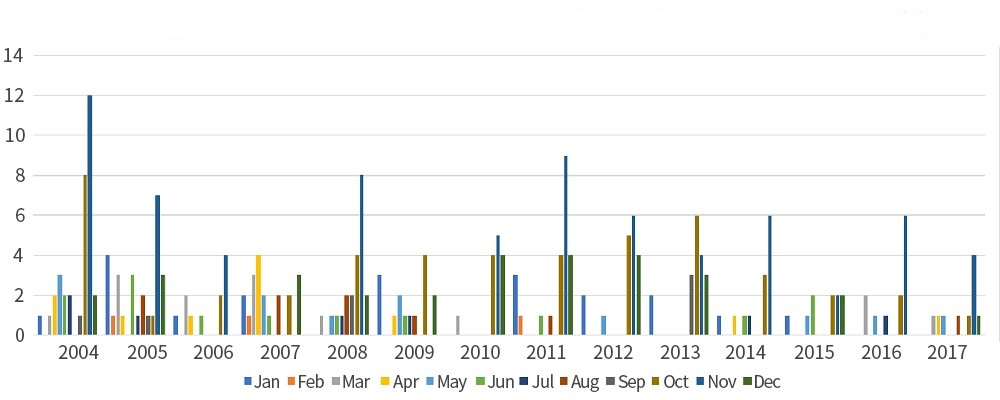


Figure S4. The number of hemorrhagic fever deaths per month from 2004 to 2017.


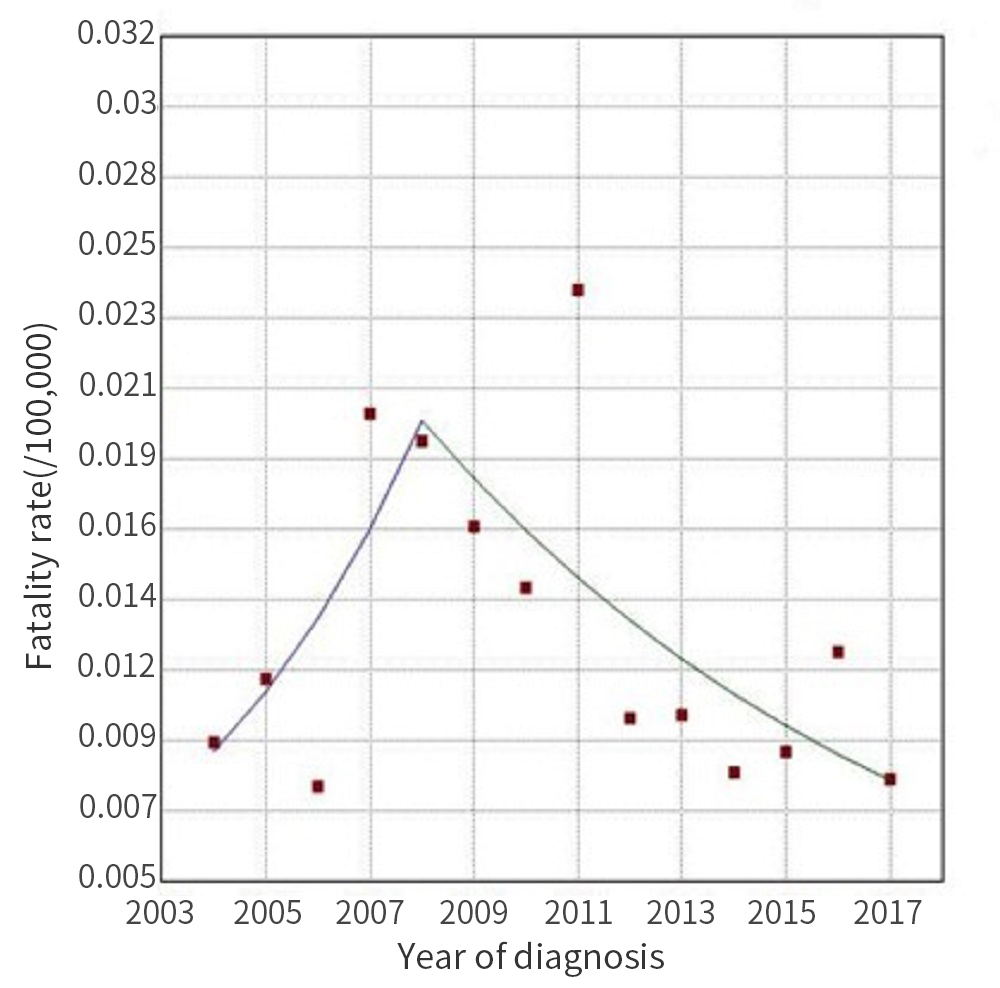


Figure S5. Trends in the fatality rate of HFRS from 2004 to 2017 shown by Joinpoint regression analysis. From 2004 to 2008, the annual fatality rate increased by 21.92% (p>0.05). From 2008 to 2017, the annual fatality rate decreased by 9.55% every year (p>0.05).


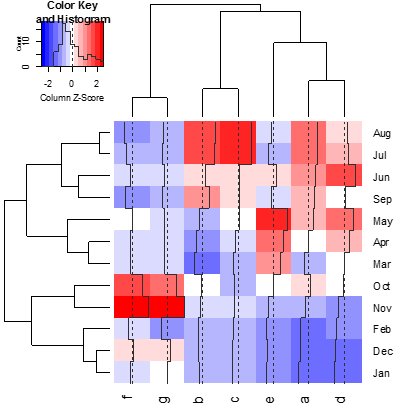


Figure S6. We conducted z-score normalization processing on the above data. Heat maps show that October and November join together in one category. The number of cases and deaths are combined.


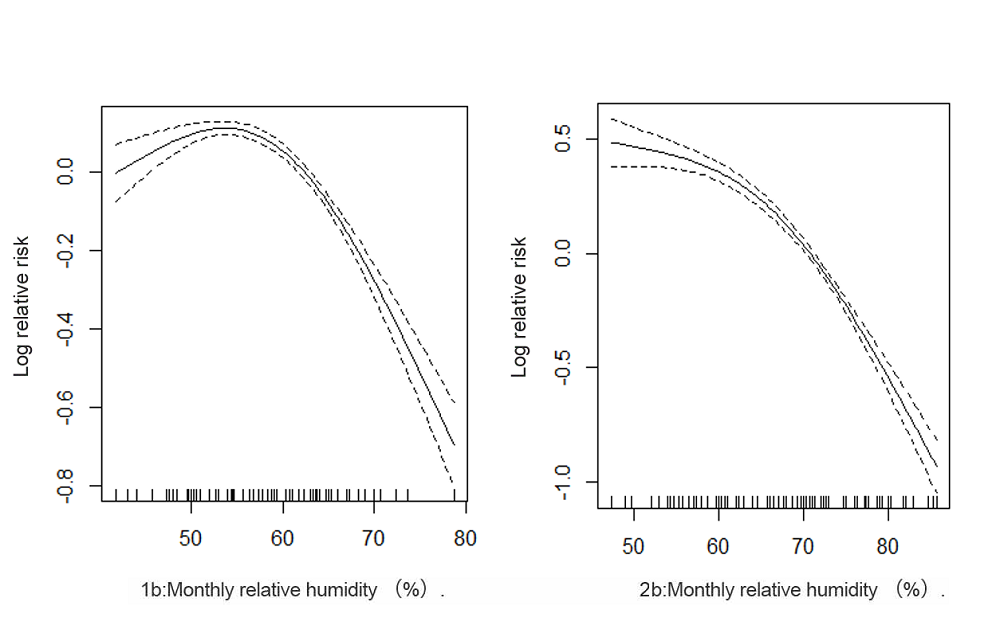


Fig. S7 The relationship between monthly relative humidity and the incidence of HFRS.


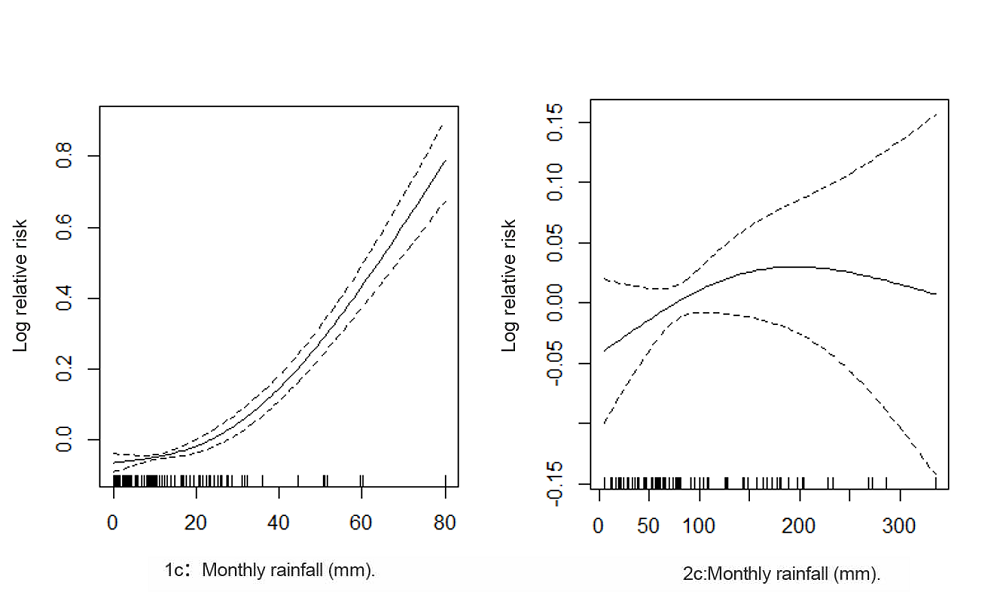


Fig. S8 The relationship between monthly rainfall and the incidence of HFRS.


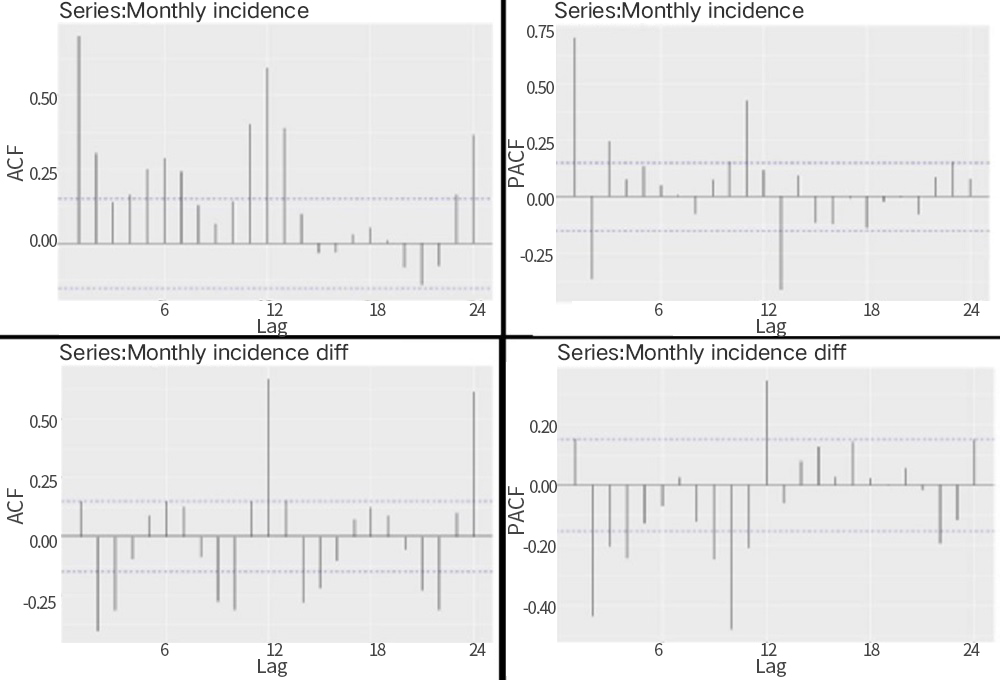


Figure S9. Sequence of hemorrhagic fever and differential ACF and partial autocorrelation coefficient (PACF) charts from 2004 to 2017.


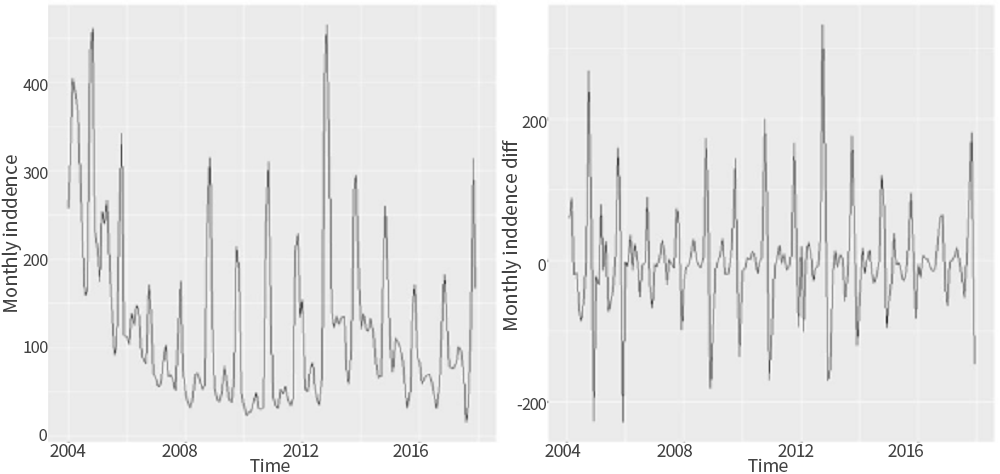


Figure S10-f1. Timing chart of the number of hemorrhagic fever cases from 2004 to 2017 in Shandong province.

Figure S10-f2. First-order differential timing diagram of the number of hemorrhagic fever cases from 2004 to 2017 in Shandong province.


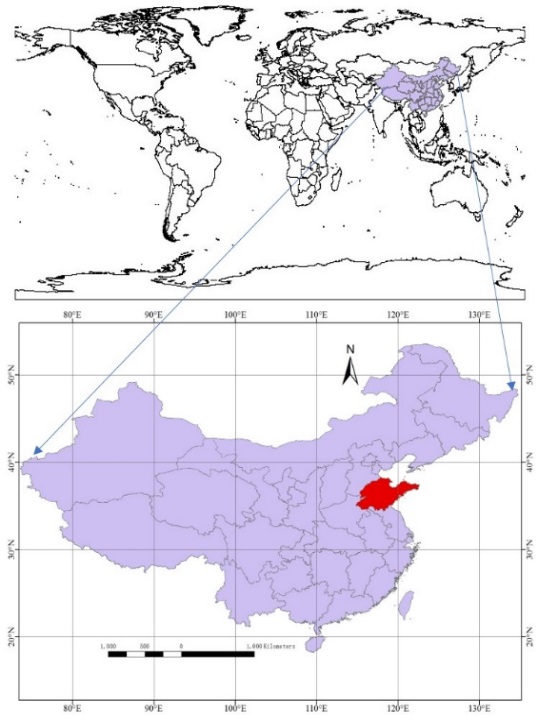


Figure S11 The red area indicates the geographical location of Shandong province.
